# Tissue detection of natural killer cells in colorectal adenocarcinoma

**DOI:** 10.1186/1471-230X-4-20

**Published:** 2004-09-13

**Authors:** Ioannis S Papanikolaou, Andreas Ch Lazaris, Periklis Apostolopoulos, Nikos Kavantzas, Maria G Papas, Christos Mavrogiannis, Efstratios S Patsouris, Athanasios Archimandritis

**Affiliations:** 1 Department of Gastroenterology, H. Venizelou General Hospital, Faculty of Nursing, National University of Athens, Athens, Greece; 2 Department of Pathology, National University of Athens Medical School, Athens, Greece; 3 Department of General Medicine, University Hospital, Dijon, France; 4 Department of Pathophysiology, Gastroenterology Section, National University of Athens Medical School, Athens, Greece

## Abstract

**Background:**

Natural killer (NK) cells represent a first line of defence against a developing cancer; however, their exact role in colorectal cancer remains undetermined. The aim of the present study was to evaluate the expression of CD16 and CD57 [immunohistochemical markers of natural NK cells] in colorectal adenocarcinoma.

**Methods:**

Presence of NK cells was investigated in 82 colorectal adenocarcinomas. Immunohistochemical analysis was performed, using 2 monoclonal antibodies (anti-Fc Gamma Receptor II, CD16 and an equivalent to Leu-7, specific for CD-57). The number of immunopositive cells (%) was evaluated by image analysis. The cases were characterized according to: patient gender and age, tumor location, size, grade, bowel wall invasion, lymph node metastases and Dukes' stage.

**Results:**

NK cells were detected in 79/82 cases at the primary tumor site, 27/33 metastatic lymph nodes and 3/4 hepatic metastases; they were detected in levels similar to those reported in the literature, but their presence was not correlated to the clinical or pathological characteristics of the series, except for a negative association with the patients' age (p = 0.031).

**Conclusions:**

Our data do not support an association of NK cell tissue presence with clinical or pathological variables of colorectal adenocarcinoma, except for a negative association with the patients' age; this might possibly be attributed to decreased adhesion molecule expression in older ages.

## Background

Colorectal adenocarcinoma is a neoplasm in which prognosis is mainly determined by the histological stage. However, the prognosis of certain patient groups, especially those of intermediate stages, remains vague [1]. The main weakness of the currently used prognostic markers in colorectal adenocarcinoma, is their inability to point out patients without metastases, whose clinical course will progress unfavorably and patients with metastatic disease who will have a relatively better outcome [1]. Therefore, development of new prognostic markers is essential, as they might help in the planning of more effective treatment modalities. Markers of the host's immune response could potentially be helpful in this field.

Natural killer (NK) cells play a pivotal role in innate immunity and immunological surveillance. Their cytotoxic effects may be initiated without prior immunization and thus NK cells have been considered as a first line of defence of the host against a developing cancer [2,3]. In spite of their small presence in tissue samples from colorectal adenocarcinomas, NK cells manifest a potent cytotoxic anti-tumor effect [3-5]. Expression of various protein surface markers is essential for these cells to perform their activities [2]. Among these markers, CD16 is used to identify active NK cells in immunohistochemical studies, while CD57 is expressed on NK cell-like elements.

The aim of this study was to evaluate tissue presence of NK cells in a series of colorectal adenocarcinomas and to attempt to correlate their presence with clinical and pathological variables and prognostic markers of colorectal cancer.

## Methods

From June 1997 to May 2000, 82 patients from our Department underwent colectomy, due to a diagnosis of colorectal adenocarcinoma of conventional histologic type. NK cell presence was examined via standard 3-step immunohistochemical analysis (ABComplex), which was performed on formalin-fixed, paraffin-embedded tissue sections. Briefly, two appropriate monoclonal antibodies were used (anti-Fc Gamma Receptor II, CD16 and an equivalent to Leu-7, specific for CD-57, Dako, Glostrup, Denmark) at dilutions of 1/200 with overnight incubation. Antigen retrieval was necessary and was performed by usual microwave treatment. In the examined samples, tissue identification of NK cells was based on strong CD16 immunostaining, which was frequently accompanied by CD57 immunoreactivity. Tissue sections from hypertrophic tonsils were used as positive markers. Diaminobenzidine tetrahydrochloride 0.06% in phosphate-buffered saline buffer containing 0.03% hydrogen peroxide was used as a chromogen. Images were acquired using a Zeiss Axiolab microscope (Carl Zeiss GmbH, Jena, Germany) with a mechanical stage, fitted in a Sony-iris CCD video camera (Sony, Tokyo, Japan). The video camera was connected to a Pentium II PC, loaded with the appropriate image analysis software (Sigma Scan Pro, Science, Erkrath, Germany). Slides were examined at a ×200 magnification. The ratio, expressed in percentile proportion (%), between the number of immunohistochemically positive-stained cells and the total number (stained and unstained) of lymphocytes was calculated. All tumors had been characterized according to the following classical clinical and pathological variables: patient gender (male 52, female 30, 63.4% and 36.6%, respectively) and age (mean: 75.9, median: 76, ranging between 35–95 years), tumor location (rectum 27, 32.9%, sigmoid 26, 31.7%, descending colon 4, 4.8%, transverse colon 5, 6%, ascending colon 10, 12.1%, cecum 10, 12.1%), size (mean diameter: 4.4 cm, median diameter: 4 cm, ranging between 1.2–10 cm), grade (I: 24 cases, 29.3%, II: 48 cases, 58.5%, III: 10 cases, 12.2%), bowel wall invasion (present in 66 cases, 80.5%), lymph node metastases (present in 33 cases, 40.2%) and Dukes' stage (A: 5, 6.1%, B: 44, 53.7%, C: 29, 35.4%, "D": 4, 4.9%). All "D" Dukes' stage cases involved hepatic metastases. The tumors were categorized according to their location into two larger groups: those involving the rectum as well as the left colon (57 cases, 69.5%) and those involving the right colon (25 cases, 30.5%). This was performed for reasons of statistical analysis.

Moreover, positive cases were divided into two groups: those in which immunopositive cells ranged from 0–9% of the total number of lymphocytes ("weak" tissue presence of NK cells) and those in which they were ≥10% ("strong" NK cell presence). This was performed somewhat arbitrarily, as we considered that the cutoff level of 10% is in accordance to what a pathologist would consider to be the cutoff level between "negative" and "positive" cases in a qualitative examination, and in order to perform statistical analysis. Associations of NK cell presence with the patients' sex, tumor location, grade, and presence of bowel wall invasion, as well as lymph node metastases and Dukes' stage were examined using chi-square statistics; associations with the patients' age and tumor size were determined using the Mann-Whintey U test.

## Results

NK cells were detectable in the primary tumor site of 79 cases (96.3%); their percentages were up to 32% of the primary site lymphocytes (Figure). Of the 33 cases with metastatic lymph nodes, NK cells were found in 27 cases (81.8%); moreover, NK cells were found among liver lymphocytes in 3 of the total 4 cases with hepatic metastases (75% of stage "D" cases).

**Figure 1:**
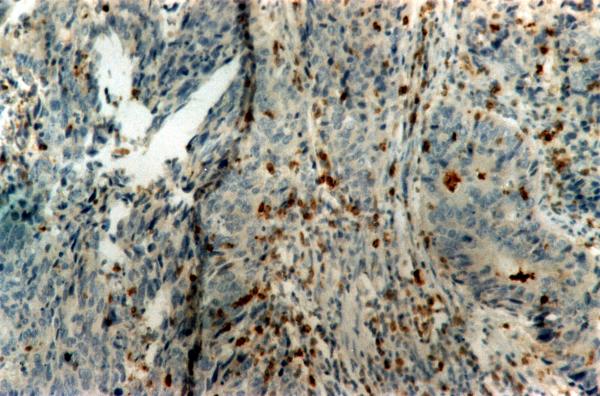
Presence of NK cells within cancerous formations. (ABC, magnification ×250).

Forty-nine of the 82 primary tumors (59.8%) had a "weak" tissue presence of NK cells (0–9%), whereas the remaining 33 primary tumors (40.2%) had a "strong" NK cell presence (≥10%). Of the 33 cases with metastatic lymph nodes, 18 cases (54.5%) had "weak" and 15 "strong" NK cell presence.

NK cell presence in the primary tumor site was not associated with the patients' sex (chi-square, p = 0.462), tumor size (Mann-Whintey U test, p = 0.377), tumor location (chi-square, p = 0.262), tumor differentiation (chi-square, p = 0.556), wall invasion (chi-square, p = 0.999), the presence of metastatic lymph nodes (chi-square, p = 0.720), or Dukes' stage (chi-square, p = 0.992). However, a negative association between the presence of NK cells at the primary tumor site with the patients' age was noticed (Mann-Whitney U test, p = 0.031).

Moreover, the presence of NK cells in the metastatic lymph nodes was not associated with the patients' sex (chi-square, p = 0.999), age (Mann-Whintey U test, p = p = 0.212), tumor size (Mann-Whintey U test, p = 0.729), tumor location (chi-square, p = 0.442), tumor differentiation (chi-square, p = 0.872), wall invasion (chi-square, p = 0.999), or Dukes' stage (C or D) (chi-square, p = 0.308).

## Discussion

Although colorectal adenocarcinoma has been characterized as a model neoplasm in which clinical prognosis is mainly determined by the histological stage, pathologic markers often fail to predict the prognosis of a great number of patients. This has necessitated the evaluation of several alternative prognostic markers [1]. Immunohistochemical studies have proved useful in the evaluation of such potential markers; although they are relatively cheap and easy-to-perform, they may be as effective as other advanced molecular techniques in determining the role of several molecules in human carcinogenesis [6].

CD16 and CD57 were chosen in the present study, as they are markers of the presence of NK cells, a cellular line which could reasonably play a significant role in the pathophysiology of colorectal adenocarcinoma. NK cells were considerably present in the primary tumor site, as well as the metastatic lymph nodes of many cases in our series. This observation is in line with data deriving from the literature [7]. NK cell presence had no correlation with the clinical or pathological variables of our series, besides a negative association between their presence at the primary tumor site and the patients' age (p = 0.031), i.e. less NK cells were found in the stroma of the primary tumor site in the older patients of our study. Reports concerning alterations of the number and/or the cytotoxicity of NK cells in patients of older ages seem to be controversial [8-14]; this has been attributed to the diversity of the parameters examined in these studies [8]. In most reports, advanced age results in an increase of the number of circulating NK cells [8-10]; however, other studies point out that although total levels of NK cells actually remain steady, some of their biologically active sub-populations actually diminish [11,12]. The cytotoxicity of NK cells on the other hand, has been reported to increase [13], remain steady [8,14] or diminish [12] in patients of older age; some reports connect presence of certain nutrients and hormonal factors with the maintenance of NK cytotoxicity in these patients [8,14].

However, what seems to be pivotal for rapid and efficient migration of NK cells from the circulation to the tumor stroma, is the expression of appropriate adhesion molecules [15]. As expression of adhesion molecules in lymphocytes, monocytes and the interstitial tissue decreases with age [16] this might result in decreased adhesion molecule-mediated migration of NK cells to the tumor stroma in the older-aged patients of our series; this could possibly explain the negative association observed in our study [5,15].

In conclusion, our findings suggest that less NK cells are found in the stroma surrounding the primary tumor site in older patients with colorectal adenocarcinoma. This could possibly be attributed to decreased adhesion molecule-mediated migration; however, this hypothesis needs to be further investigated through more studies.

## Pre-publication history

The pre-publication history for this paper can be accessed here:


